# Corroborating written history with ancient DNA: The case of the Well-man described in an Old Norse *saga*

**DOI:** 10.1016/j.isci.2024.111076

**Published:** 2024-10-25

**Authors:** Martin R. Ellegaard, S. Sunna Ebenesersdóttir, Kristjan H.S. Moore, Anna Petersén, Åshild J. Vågene, Vanessa C. Bieker, Sean D. Denham, Gianpiero L. Cavalleri, Edmund Gilbert, Thomas Werge, Thomas F. Hansen, Ingrid Kockum, Lars Alfredsson, Tomas Olsson, Eivind Hovig, M. Thomas P. Gilbert, Kári Stefánsson, Hans K. Stenøien, Agnar Helgason, Michael D. Martin

**Affiliations:** 1 Department of Natural History, NTNU University Museum, Norwegian University of Science and Technology, Trondheim, Norway; 2 Section for Hologenomics, Globe Institute, Faculty of Health and Medical Sciences, University of Copenhagen, Copenhagen, Denmark; 3 deCODE genetics/AMGEN, Inc., Reykjavik, Iceland; 4 Department of Anthropology, University of Iceland, Reykjavik, Iceland; 5 Department of Archaeology, Norwegian Institute of Cultural Heritage Research, Trondheim, Norway; 6 Museum of Archaeology, University of Stavanger, Stavanger, Norway; 7 School of Pharmacy and Biomolecular Sciences, Royal College of Surgeons in Ireland, Dublin, Ireland; 8 The Lundbeck Foundation Initiative for Integrative Psychiatric Research, iPSYCH, Aarhus, Denmark; 9 GLOBE Institute, Center for GeoGenetics, University of Copenhagen, Copenhagen, Denmark; 10 Institute of Biological Psychiatry, MHC Sct. Hans, Mental Health Services Copenhagen, Roskilde, Denmark; 11 Department of Clinical Medicine, University of Copenhagen, Copenhagen, Denmark; 12 Danish Headache Center, Department of Neurology, Rigshospitalet-Glostrup, Glostrup, Denmark; 13 Novo Nordisk Foundation Center for Protein Research, Translational Disease Systems Biology, University of Copenhagen, Copenhagen, Denmark; 14 Department of Clinical Neuroscience, Karolinska Institutet, Stockholm, Sweden; 15 Center for Molecular Medicine, Stockholm, Sweden; 16 Institute of Environmental Medicine, Karolinska Institutet, Stockholm, Sweden; 17 Centre for Bioinformatics, Department of Informatics, University of Oslo, Oslo, Norway; 18 Department of Tumor Biology, Institute for Cancer Research, Oslo University Hospital, Oslo, Norway; 19 FutureNeuro SFI Research Centre, Royal College of Surgeons in Ireland, Dublin, Ireland

**Keywords:** Paleobiology, Paleogenetics, Archeology, History

## Abstract

The potential of ancient DNA analyses to provide independent sources of information about events in the historical record remains to be demonstrated. Here we apply palaeogenomic analysis to human remains excavated from a medieval well at the ruins of Sverresborg Castle in central Norway. In *Sverris Saga*, the Old Norse *saga* of King Sverre Sigurdsson, one passage details a 1197-CE raid on the castle and mentions a dead man thrown into the well. Radiocarbon dating supports that these are that individual’s remains. We sequenced the Well-man’s nuclear genome to 3.4× and compared it to Scandinavian populations, revealing he was closely related to inhabitants of southern Norway. This was surprising because King Sverre’s defeated army was assumed to be recruited from parts of central Norway, whereas the raiders were from the south. The findings also indicate that the unique genetic drift seen in present-day southern Norwegians already existed 800 years ago.

## Highlights

• Reports the first genome sequence of a character described in an Old Norse *saga*
• Confirms the remains are from a male with ancestry from southern Norway
• Isolation and differentiation of southern Norwegians was already present 800 years ago
• New information about an event only partly documented in the historical record

## Graphical abstract

**Figure:**
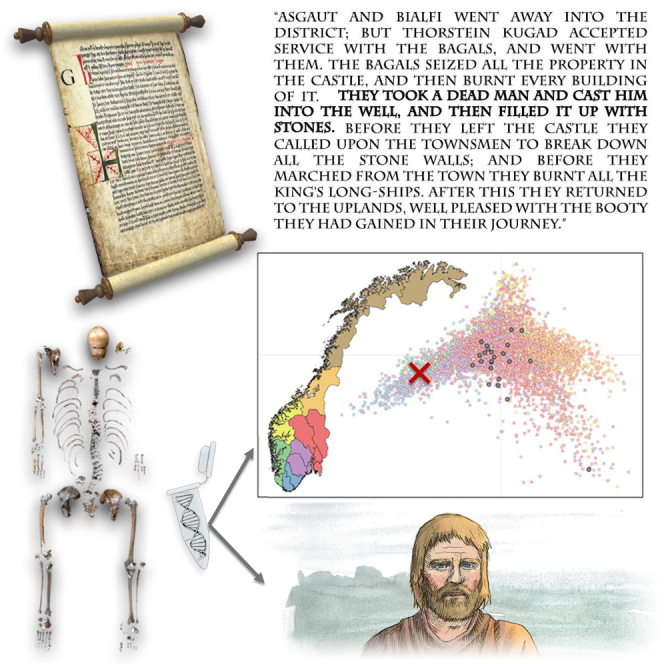

## Introduction

The Old Norse *sagas* illustrate the development of power dynasties, the reign of the Norwegian kings and important political events in medieval Norway (c. 1060–1537 CE). Largely composed by Icelandic scholars, the *sagas* were written centuries later than the events they described and were likely built on oral traditions and earlier lost manuscripts.^1^^,^^2^^,^^3^ One of these, *Sverris Saga*, concerns the Norwegian king Sverre Sigurdsson (1151–1202 CE) and describes his ambitious rise to power as sovereign over Norway in the second half of the 12^th^ century. Much of Norway’s early history is known from this single text, which depicts a period of political instability characterized by conflicts and civil wars lasting more than a century (1130–1240 CE). These wars were largely caused by disputes about succession to the throne, wherein Sverre emerged as one of the contenders, based on his claim of being a son of King Sigurd Munn, who was slain by his brother in 1155 CE.

It is widely thought that most of the *saga* text was written contemporaneously with the described events and by someone close to the king, possibly the Icelandic Abbot Karl Jónsson, at the request of Sverre and under his scrutiny.^4^^,^^5^ The text, clearly in favor of Sverre, is rich in names, places, and events, spanning an impressive 182 verses, and is unique in its detailed depiction of the many battles, and recounting a large cast of individuals, military strategic considerations, and the many speeches made by Sverre. Sverre’s men were called “Birkebeiner,” meaning “birch legs,” allegedly named so as they used primitive birch bark as leg- and footwear. His opponents, coordinated by the national representatives of the Roman Catholic Church, were called the “Bagleres” from the Norse word *bagall*, meaning “bishop wand.”

A specific passage in *Sverris Saga*^6^ describes in detail how in 1197 CE, while King Sverre wintered in Bergen, the Baglers launched a sneak attack against the Birkebeiner stronghold at Sverresborg Castle built by Sverre around 1180 CE (63° 25′ 10.1922″, 10° 21′ 25.4298″) just west of Nidaros (now the present-day city of Trondheim, Norway). The Bagler army entered the castle through a secret door while the residents were dining. They plundered and raided the castle, burning every house inside, sparing the residents only the clothes they were wearing. Crucially for this study, they threw a dead man’s body down the local drinking well inside the castle, subsequently filling it with boulders.^6^

**Figure 1:**
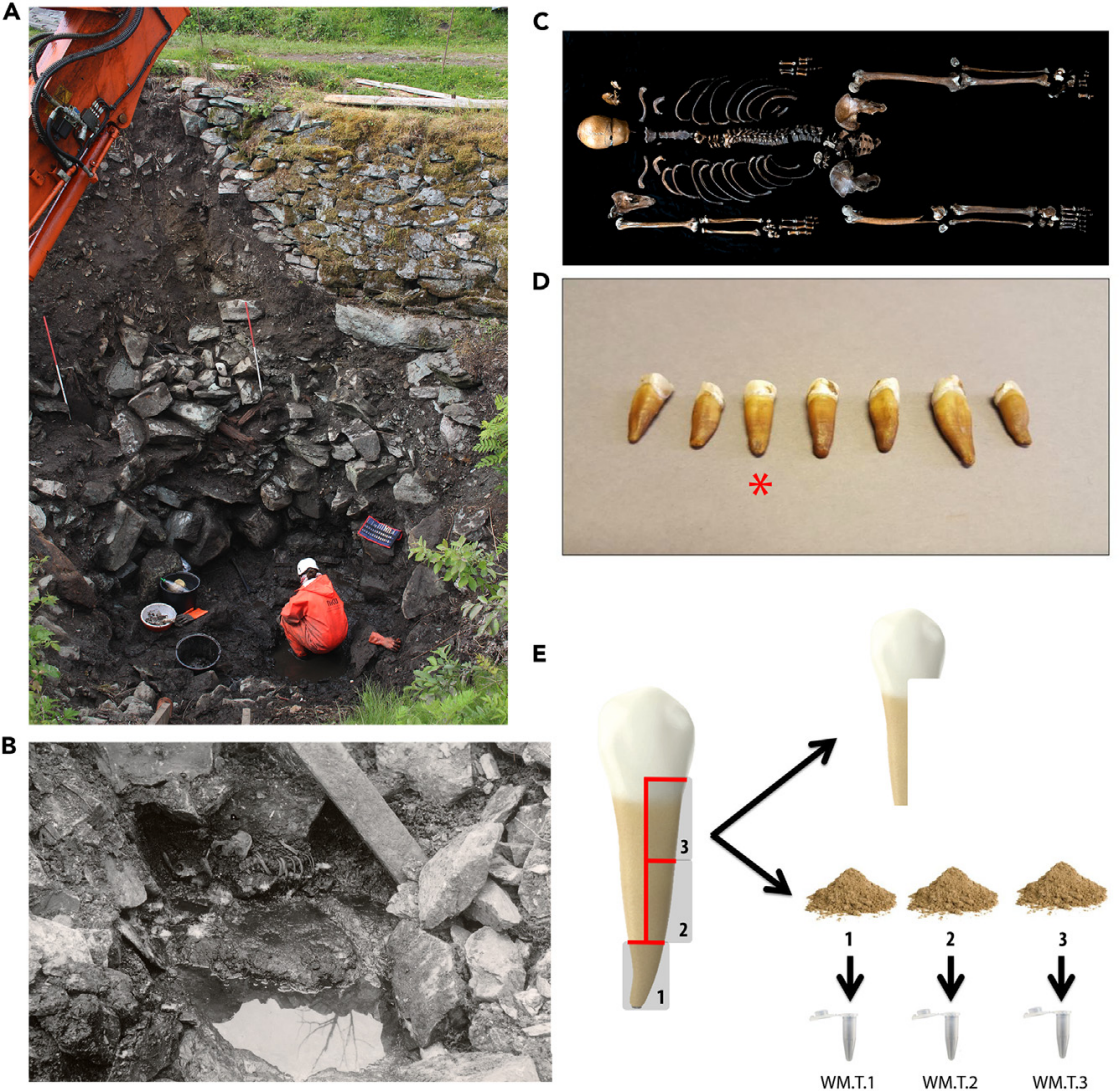
Excavation and sampling of skeletal remains from the Sverresborg Well-man (A) Photograph of the skeletal remains during a 2016 excavation. Photo courtesy of the Norwegian Institute for Cultural Heritage Research. (B) Photograph of the skeletal remains during the 1938 excavation. Photo courtesy of Riksantikvaren. (C) Photograph of the complete skeletal remains. Photo courtesy of Åge Hojem, NTNU University Museum. (D) Photograph of the seven teeth recovered, with the tooth used for ancient DNA analysis indicated by a red star. (E) Schematic illustration of the experimental design for the ancient DNA extractions. Red lines indicate cut sites along the tooth.

Early, incomplete, excavations of the well in 1938 unearthed a body of an individual at the base of the well below a substantial aggregation of large stones (Figure 1). New excavations were performed in 2014 and 2016,^7^^,^^8^ and in the southern side of the well additional worked and unworked stones were found, partly sealing new parts of the body not identified in 1938 (Figure S1). The osteological analyses from 2014 to 2016 indicate the remains belonged to a male, aged 30–40 years at the time of death.^8^^,^^9^ A photograph from the 1938 excavation shows the torso belonging to a human skeleton leaning slightly on its left side (Figure 1). Investigations in 2016^8^ revealed additional important details. The left arm was missing, but phalanges belonging to the left hand were found *ex situ*. The skull was also found *ex situ*, to the right of the upper part of the torso, and was not connected to the body. The skeleton displays several traumas, but due to the conditions, it has been difficult to differentiate which of these are *antemortem* or *postmortem*. A blunt force injury to the rear left part of the skull in addition to two sharp force cuts in the skull are not likely *postmortem* events.^8^

Radiocarbon dating of bone from the skeleton produced a conventional radiocarbon age of 940 ± 30 years (Beta-394180, bone).^10^ We sought to shed further light on the Well-man and the events described in *Sverris Saga*, by sequencing his genome and making inferences about his sex, ancestry, and physical characteristics. Our study provides an unusual opportunity to gain a deeper understanding of this historical event through the integration of results from isotopic, osteological, archaeological and genetic analyses in the context of information from an 800-year-old text.

## Results

### Calibration/correction of radiocarbon date range

Stable isotope ratio mass spectrometry measurements of ^13^C and ^15^N isotopes obtained from a sample of bone from the skeleton (δ^13^C = 19.84‰, δ^15^N = 12.38‰, TRa-13485) were used to estimate a marine dietary component of 20%. The conventional radiocarbon age of 940 ± 30 was then calibrated and corrected for the marine reservoir effect using IntCal20^11^ and Marine20,^12^ based on a 20% marine dietary input and no local reservoir offset. The resulting calibrated/corrected date range, 1055–1076 (2.5%), 1153–1277 (92.9%) cal CE, agrees well with the expected date of the Sverresborg Castle raid, 1197 CE.

### Authenticity of sequencing data

We performed DNA extractions from three different 50-mg portions of a single premolar tooth from the Well-man’s mandible, denoted WM.T.1, WM.T.2, and WM.T.3 (Figure 1E). Each of these three DNA extracts were divided into two aliquots, where one was USER-treated (denoted U), while the other aliquot was not (denoted N) in order to characterize the aDNA damage pattern. All six aliquots were used to prepare sequencing libraries, resulting in a total of six libraries, all of which yielded human genomic DNA. Only WM.T.1.U and WM.T.1.N yielded sufficient endogenous DNA and library complexity to make further sequencing feasible (Figure S2) aiming to obtain >1× average depth-of-coverage. All libraries contained highly fragmented DNA, with a mean of 47.9 bp and a mode of 30 bp for the length of mapped reads (Figure S2). The sequencing resulted in a total of 5,860,146,617 read pairs (150 PE), of which 204,791,462 mapped uniquely to the human reference genome (3.5% endogenous content). After quality filtering, this resulted in an average of 3.4× depth per position in the autosomal genome (Table S1).

The three non-USER treated DNA extracts showed signs of DNA degradation with cytosine deamination rates varying between 4% and 7% for the first 5′-end nucleotide position (Figure S3). The cytosine deamination signal was reduced to 1.3%–2.8% in the USER-treated samples. None of the six libraries showed strong evidence of contamination based on reads mapped to mtDNA (0.1%–3.2%) or the X chromosome (0.008% using the conservative “method 1” in analysis of next generation sequencing data (ANGSD), *p* value = 0.6226 from a Fisher’s exact test) (Table S2). Human contamination of the DNA extraction blank and library no-template control (NTC) was negligible (Table S1).

### Karyotype determination and uniparental marker analysis

We assign the Well-man an XY karyotype (male) based on the ratio of reads mapped to the sex chromosomes, with 5,302,575 reads mapping to the X chromosome and 515,795 mapping to the Y chromosome, yielding an R_Y_ ratio of 0.0886 with 95% confidence interval of 0.0884–0.0889 (Table S3). Confident XX assignment is R_Y_ < 0.016 while confident XY assignment is R_Y_ > 0.075.^13^

A total of 492,422 reads mapped uniquely to the rCRS mitochondrial reference sequence, yielding an average depth of coverage of 1,279×. The mitochondrial haplogroup was identified as H2a2a1, which is primarily found in Scandinavia and Eastern Europe.^14^^,^^15^^,^^16^ The Y chromosome was sequenced to an average depth of 0.78×, with the haplogroup assigned to I1a1a3a1, which is almost exclusively found in present-day Scandinavia.^17^^,^^18^

### Ancestry and phenotype inference

**Figure 2:**
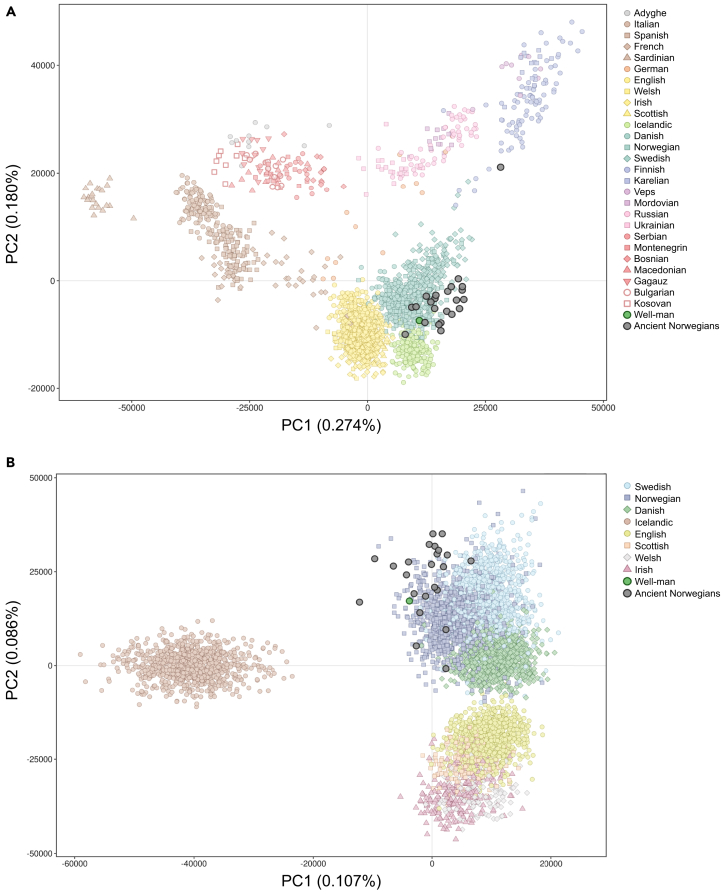
The Sverresborg Well-man is highly related to ancient and present-day Norwegians (A) Scatterplot of the first two PCs from a PCA highlighting the genetic differences between 2,139 present-day individuals from 27 European populations, with the projected coordinates of the Well-man and 23 other ancient Norwegians. (B) The Well-man and 23 ancient Norwegians projected onto the first two PCs of a PCA of 5,470 present-day individuals from Scandinavia, the British Isles, and Iceland.

Figure 2 displays two PCAs using present-day individuals from Europe (Figure 2A) and northwestern Europe (Figure 2B) along with the projected coordinates of the Well-man and other contemporaneous ancient DNA samples from Norway. The Well-man sample is located on the outermost edge of a cluster comprising the present-day Norwegian population and within the diversity of other ancient genomes from Norway. A supervised run of admixture (*K* = 2) testing for Norse (Norway and Sweden) versus Gaelic (Scotland and Ireland) ancestry^19^ revealed the Well-man to be 100% Norse.

**Figure 3:**
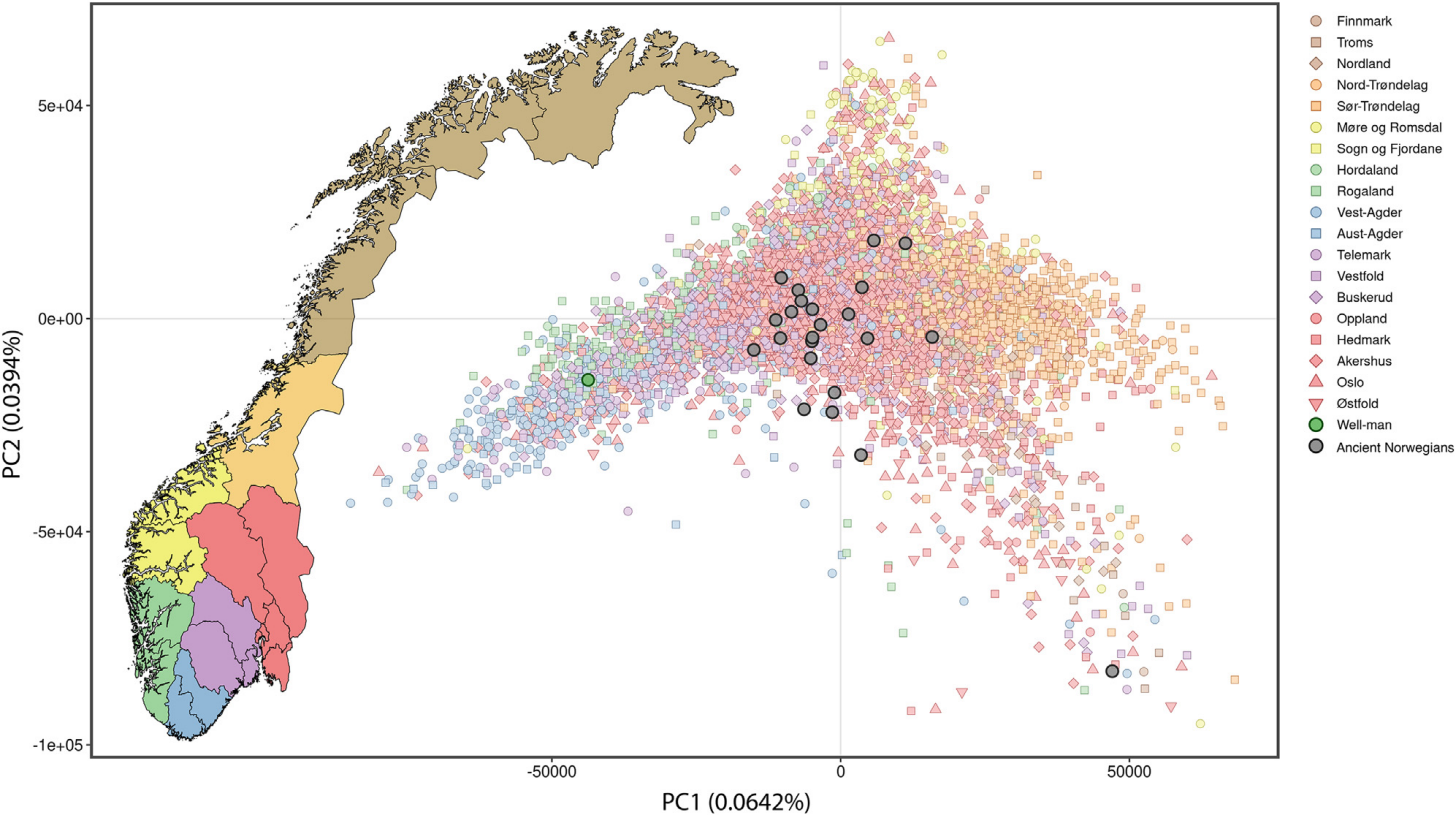
Map of Norway with color-coded counties and principal component analysis of 6,140 present-day Norwegian individuals with assigned regional associations as shown in the map, as well as 24 ancient Norwegian individuals The ancient outlier VK519 (yellow circle, lower right corner) has substantial Saami ancestry.

To determine whether the Well-man’s ancestry can be assigned to a particular sub-region of Norway, we used a principal-component analysis (PCA) based on 6,140 present-day Norwegians from 19 different counties.^20^ The Well-man is projected closest to populations from the southernmost counties of Agder, Rogaland, and Telemark based on the first two PCs (Figure 3). To further verify this result, we calculated the Euclidean distances between each ancient sample (see following text) and all present-day reference individuals using the first ten PCs (weighted by their eigenvalues) and found the Well-man to be closest to present-day Norwegians from Vest-Agder in PCA space and significantly (t test *p* < 1 × 10^−10^) closer than to any other county of Norway, except Aust-Agder, where the difference is not statistically significant (Table S4).

While the present-day genetic structure of the Norwegian regions is evident (Figure 3), it may have been different 800 years ago, when the Well-man lived. For example, it is theoretically possible that the pattern of ancestry that now distinguishes people from Southern Norway was more widespread in the past. To confidently and exclusively assign the ancestry of the Well-man to southern Norway, it is necessary to show that individuals with northern Norwegian ancestry also existed at that time in and around Trondheim. To examine this, we obtained previously published sequence data from 23 ancient Norwegians (20 from a study by Margaryan et al.^21^ and three from the central and northern regions of Norway^22^ and securely dated to a similar time period as the Well-man) (Table S5). Using genotypes imputed in the same fashion as for the Well-man, PCA projection shows that these ancient Norwegians are more similar to present-day individuals from central Norway, where they were excavated, than to present-day southern Norwegians and are quite distinct from the Well-man (Figure 3). We note one outlier (VK519) that was previously found to carry substantial Saami ancestry.^21^ This demonstrates that the genetic differences between northern and southern Norwegians observed today were at least partially in place 800 years ago and confirms that the ancestry of the Well-man was indeed from southern Norway.

**Figure 4:**
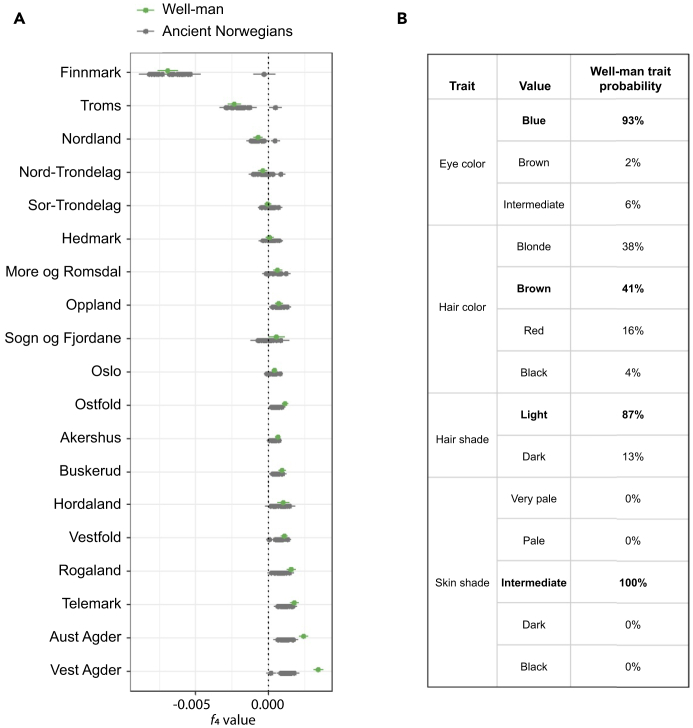
Ancestry and phenotype inferences for the Sverresborg Well-man (A) *f*_4_ summary statistics in the form YRI:Ancient; Danish:X. The y axis is formatted according to the geographical distribution of the regions in Norway (southern regions at the bottom). Error bars indicate 1 SE. (B) HIrisPlex predictions of trait probabilities for the Well-man. Traits values of highest probability are indicated in bold.

These results are supported by *f*_4_-statistics using the Well-man and each of the Norwegian regions shown in Figure 3. The *f*_4_-statistics are calculated as YRI:Ancient; Danish:X, where Ancient represents the Well-man or one of the other ancient Norwegians and X represents the present-day inhabitants of a Norwegian county (Figure 4). Positive *f*_4_-values indicate affinity between Ancient and X. The results are consistent with PCAs, and we note the Well-man has noticeably higher *f*_4_ values for Vest-Agder and Aust-Agder than any other ancient individual.

A recent study of population structure in present-day Norway showed that in addition to being differentiated due to genetic drift, inhabitants of the Agder counties are also characterized by relatively high levels of inbreeding.^20^ As a matter of curiosity, we therefore estimated the degree of inbreeding in the Well-man and the other ancient Norwegians. This revealed that the Well-man has an inbreeding coefficient of 0.0114, with a rather large 19 cM homozygous fragment on chromosome 18 (Figure S4). However, as two of the other 23 ancient Norwegians had higher levels of inbreeding, it cannot be stated that the Well-man was necessarily unusual in this regard (Table S6).

Among the set of imputed genotypes for the Well-man were 40 that are associated with hair, eye, and skin color (Table S7). Using the HIrisPlex prediction model for pigmentation phenotypes,^23^^,^^24^^,^^25^ we find that the Well-man likely had blue eyes, fair skin tone, and blond or light brown hair color (Table S7; Figure 4B). The screening of all six libraries for the presence of acquired pathogens (non-commensal taxa known to cause infectious disease) revealed no reliable hits.

## Discussion

When knowledge of the past is primarily based on historical texts such as *Sverris Saga*, it is valuable to have independent sources of information that can be used to verify, alter or add to our understanding of protagonists and events. Genetic data from ancient remains can be an important new source of information in such instances. In particular, whole-genome sequence data allow unprecedented inferences about individuals recovered from archaeological excavations, such as their sex, physical characteristics, kinship, and ancestry.

The Norse and Icelandic *sagas* provide a rich vein of information that permeates our understanding of events in the North Atlantic region during the Viking and Medieval period. In particular, *Sverris Saga* is an important source of information about historical events in Norway during the late 12th and 13th centuries. The discovery of the remains of the Well-man and their connection to the passage in *Sverris Saga* about the raid of Sverresborg Castle in 1197 CE provides a unique opportunity to bring inferences based on ancient DNA to bear on the interpretation of historical events. While we cannot prove that the remains recovered from the well inside the ruins of Sverresborg Castle are those of the individual mentioned in *Sverris Saga*, the circumstantial evidence is consistent with this conclusion.

Our genomic analyses confirm that the Well-man was indeed a male and augment the historical text with predictions about his physical appearance: that he likely had blue eyes and blond or light-brown hair. More interestingly, our results show that his ancestry can be traced to the southernmost counties of Norway, most probably Vest-Agder. The defeated holders of the castle were King Sverre’s Birkebeiners, who are thought to have been mainly from central Norway. Conversely, it is the Baglers, the invading victors from the south of Norway, who are described as having thrown the man into the well. Accordingly, previous reports had assumed that the Well-man was a central Norwegian, from the losing side of Birkebeiners.^10^^,^^26^ Our results unequivocally show that the Well-man’s ancestry was typical of the present-day population of the southern Agder counties, but of course cannot tell us whether the Well-man belonged to the Birkebeiner or the Bagler army. We note that the passage in *Sverris Saga* states that the Well-man was dead before the Baglers threw him into the well. Perhaps the Baglers threw one of their own dead into the well.

While the intent of the Baglers is not known for certain, the *Sverris Saga* text indicates that they aimed to render the castle undefendable and uninhabitable for King Sverre and his followers. It has been speculated that throwing a body into the well was an attempt at biological warfare^26^ a corpse into the only stable, nearby source of drinking water would lead to pollution of the water, an effect that could have only been amplified by a diseased corpse. Although we detected no signs of pathogens in the metagenomic sequences retrieved from the Well-man’s tooth, we note that the stringent decontamination procedures used during tooth sample preparation (removal of tooth cementum and enamel, UV irradiation) may have removed pathogen DNA and thus rendered it undetectable. Still, despite that numerous previous studies have demonstrated that pathogen DNA can be recovered from human dental pulp cavity and calculus, the lack of detection using this study’s methods does not allow us to conclude that the Well-man was not infected by microbial pathogens at the time of his death.

Arguably, the most important finding in this study is that a substantial component of the distinctive allele frequencies that currently characterize the southernmost Agder counties of Norway^20^ was already in place 800 years ago, during the reign of King Sverre. This has implications for our understanding of Norwegian population history, insofar as it implies that this region must have been relatively isolated not only since that time, but also for at least a few hundred years beforehand, and perhaps longer. The origin and persistence of this geographical stratification of the Norwegian gene pool warrant further investigation, for example through the sequencing of more ancient Norwegians from the southern counties.

### Limitations of the study

Due to issues regarding the timeline of sample availability and the need to reduce destructive sampling of the precious material, different bones were used for the radiocarbon dating and isotopic analyses. Potentially, this could introduce errors in the radiocarbon date assignment if a major change in diet occurred the last years of the Well-man’s life, thus affecting the marine reservoir effect correction of the rib bone but not the cranial vault. Another possibility, although very unlikely, is that due to varying bone turnover rates, the rib and cranial bone actually came from different individuals. Additionally, the lack of detected pathogens in the pathogen DNA analysis might be due to study design considerations rather than an actual absence of pathogen DNA.

## Resource availability

### Lead contact

Further information and requests for resources should be directed to and will be fulfilled by the lead contact, Michael D. Martin (mike.martin@ntnu.no).

### Materials availability

The Well-man archaeological remains are stored in the Norwegian University of Science and Technology (NTNU) University Museum collections with identifier N-188808.

### Data and code availability

• Raw sequencing data (FASTQ files) for the ancient Norwegian genomes used as reference material are also available on the European Nucleotide Archive, and new sequence data generated for this study have also been deposited there.
• A custom list of microbial taxa used in the pathogen screening analysis is available on GitHub.
• The human reference genome is available on the NCBI website. The 1000G phased genotypes and annotated human-genome regions of low accessibility are available from the 1000 Genomes Consortium. The human Y-DNA haplotype tree is available from the ISOGG website. The human mitochondrial genome phylogeny is available from the PhyloTree website.

## Acknowledgments

We the authors are grateful to Ellen Grav and Nina Elisabeth Valstrand for providing important logistical and technical information, and for facilitating access to the archaeological material and photographs. We also thank Axel Christophersen for useful discussion about the study's historical context and for facilitating the sampling. We gratefully acknowledge NTNU Onsager Fellowship funding to M.D.M., 10.13039/501100005416Norwegian Research Council grant 287327 to M.D.M., Carlsbergfondet Semper Ardens grant CF18-1109 to M.T.P.G., and 10.13039/501100005416Norwegian Research Council grant 262424 to Axel Christophersen. The Norwegian Institute of Cultural Research generously supported the work of A.P. by facilitating research funding.

## Author contributions

A.P., S.D.D., M.R.E., and M.D.M. performed the initial sampling. M.R.E. performed the laboratory work with contributions from V.C.B. and M.D.M. M.R.E., S.S.E., K.H.S.M., A.H., Å.J.V., V.C.B., and S.D.D. performed the computational analyses. M.R.E., A.P., A.H., and M.D.M. wrote the manuscript with contributions from all authors. H.K.S., M.D.M., and M.T.P.G. provided funding.

## Declaration of interests

The authors declare no competing interests.

## STAR★Methods

### Key resources table

**Table:** 

REAGENT or RESOURCE	SOURCE	IDENTIFIER
**Deposited data**		

Raw (FASTQ) reads	This study	E.N.A. PRJEB69227
Human reference genome NCBI build 38, hg38	Genome Reference Consortium	https://www.ncbi.nlm.nih.gov/datasets/genome/GCF_000001405.26/
20 genomes of ancient Norwegians	Margaryan et al.^21^	E.N.A. PRJEB37976
3 genomes of ancient Norwegians	Gopalakrishnan et al.^22^	E.N.A. PRJEB53899
Human genome regions of low accessibility	1000 Genomes Project	https://genome.ucsc.edu/cgi-bin/hgTrackUi?db=hg19&g=tgpPhase3Accessibility
Human genome phased genotypes (release 20201028_3202_phased)	1000 Genomes Project	https://ftp.1000genomes.ebi.ac.uk/vol1/ftp/data_collections/1000G_2504_high_coverage/working/20201028_3202_phased/
ISOGG 2019 Y-DNA Haplogroup Tree	International Society of Genetic Geneaology^27^	https://isogg.org/tree/
*PhyloTree* build 17	Van Oven and Kayser^15^	https://www.phylotree.org/tree/index.htm

**Software and algorithms**		

*PALEOMIX v. 1.2.13.4*	Schubert et al.^28^	https://github.com/MikkelSchubert/paleomix/
*AdapterRemoval v2.3.1*	Schubert et al.^29^	https://github.com/MikkelSchubert/adapterremoval
*BWA v. 0.7.10*	Lu and Durbin^30^	https://github.com/lh3/bwa
*MapDamage v. 2.0.9*	Jónsson et al.^31^	https://ginolhac.github.io/mapDamage/
*PicardTools v. 2.21.3*	Broad Institute	https://broadinstitute.github.io/picard
*BEDtools v. 2.29.2*	Quinlan and Hall^32^	https://bedtools.readthedocs.io/en/latest/
*ANGSD v.0.935*	Korneliussen et al.^33^	https://www.popgen.dk/angsd/index.php/ANGSD
*haploGrouper*	Jagadeesan et al.^34^	https://gitlab.com/bio_anth_decode/haploGrouper
*HIrisPlex*	Department of Genetic Identification of Erasmus MC	https://hirisplex.erasmusmc.nl/
*GLIMPSE*	Rubinacci et al.^35^	https://odelaneau.github.io/GLIMPSE/
*Smartpca* (*eigensoft*)	Patterson et al.^36^	https://github.com/chrchang/eigensoft/
*MALT v. 0.4.1*	Vågene et al.^37^	https://software-ab.cs.uni-tuebingen.de/download/malt/
*HOPS*	Hübler et al.^38^	https://github.com/rhuebler/HOPS
*MEGAN6*	Huson et al.^39^	https://software-ab.cs.uni-tuebingen.de/download/megan6/

### Experimental model and study participant details

This study reports on the genomic sequencing and analysis of medieval human male skeletal remains (1153-1277 cal CE, 92.9% probability) recovered during archaeological excavations at the Sverresborg Castle ruins in Trondheim, Norway. The skeletal remains are stored in the NTNU University Museum collections in Trondheim, Norway.

### Method details

#### Archaeological context

The skeletal remains of an ancient individual were discovered in 1938, in a well at the ruins of Sverresborg Castle. The remains were left largely unexcavated, and apart from a brief description^40^ and articles based on interviews with G. Fischer in the local newspaper in 1938. Unfortunately, all documentation from this excavation has subsequently been lost.^8^^,^^26^ A later excavation in 2014 recovered parts of the skeleton, while a final excavation in 2016 uncovered approximately 90% of a complete human skeleton.^8^

#### Sample tissue

After evaluation by the Norwegian National Committee for Research Ethics on Human Remains (org. 999-148-603), one premolar tooth (NTNU University Museum collection number N-188808) from the mandible was obtained from the remains to perform DNA extraction, sequencing, and analysis. One rib fragment was taken for accelerator mass spectrometry (AMS) radiocarbon dating analysis by Beta-Analytic, Inc. One sample from the frontal bone was taken for stable isotopic analysis (^13^C, ^15^N) performed on an isotope ratio mass spectrometer (IRMS) at the National Laboratory for Age Determination, NTNU. This work was undertaken as a part of the *Medieval Urban Health- From Individual to Public Responsibility, AD 1000-1600* (MedHeal600) project.

#### Calibration/correction of conventional radiocarbon age

The conventional radiocarbon age of 940 ± 30 was calibrated and corrected for the marine reservoir effect using IntCal20^11^ and Marine20.^12^ Terrestrial/marine dietary endpoints of -22‰/-12‰ for δ^13^C and 10‰/23‰ for δ^15^N were used to estimate marine dietary component. These endpoints are suggested by the latest work on calibration and correction of radiocarbon dates from human skeletal material from medieval Trondheim, which have yielded great variation and uncertainty in ΔR values estimated from skeletal material from medieval Trondheim (Seiler et al., in preparation). Thus we have chosen not to apply a local reservoir offset in the correction.

#### DNA extraction

All DNA laboratory work prior to PCR amplification was performed in a dedicated, positively pressurised ancient DNA (aDNA) laboratory facility at the University Museum, NTNU. We sampled c. 150 mg of the tooth for DNA analysis. The sample was cut into three distinct pieces and treated as individual samples throughout laboratory work to maximise yield during DNA extraction. To reduce surface contamination, samples were UV-irradiated for 30 seconds on two sides. Samples were carefully powdered to maximise the surface-volume ratio. 1 ml lysis buffer containing 0.45 M EDTA (pH 8) and 0.20 mg/ml proteinase K was added to each sample and left to incubate on a rotor for 10 minutes at 37°C to increase endogenous yield.^41^^,^^42^ After this pre-digestion step, the lysis buffer was discarded and replaced by a new lysis buffer (4 ml), and the sample was left to incubate on a rotor for 48 hours at 37°C. After cell lysis, the lysis buffer containing sample DNA was mixed in a ratio of 1:10 with a modified Qiagen PB buffer (500 ml PB buffer with 9 ml 5M NaOAc, 2 ml 5M NaCl, pH 4.5). 50 μl Silica beads were added to the solution and incubated for one hour at ambient temperature before samples were centrifuged and supernatant discarded. The pellet was resuspended in 50 μl EBT. One DNA extraction blank was included. Each of the three DNA extracts (WM.T.1, WM.T.2, and WM.T.3) was split into two aliquots denoted ‘N’ (non-USER treated) and ‘U’ (USER treated), yielding a total of six DNA aliquots. Only three of the six DNA extracts were subjected to USER treatment to reduce the characteristic damage patterns associated with DNA degradation.^43^^,^^44^^,^^45^ USER treatment was performed by adding 5 μl USER enzyme (NEB) for three hours at 37°C until finally being purified using Qiagen MinElute purification columns and eluted in 25 μl EBT.

#### Genomic library and sequencing

For each extract, 15–20 μl of extracted DNA was built into single-stranded libraries using the SCR protocol as previously described.^46^ An extraction blank and library no-template control (NTC) were also included. Each library was eluted in 30 μl EBT. Subsequently, 10 μl of each library was amplified in a 50-μl reaction for between 13 and 20 cycles, using a dual-index approach.^47^ The optimal number of PCR cycles was determined by qPCR (QuantStudio3, Thermo Fisher). The amplified libraries were purified using SPRI beads and quantified on a 2400 TapeStation instrument (Agilent Technologies) using High Sensitivity D1000 ScreenTapes. The amplified libraries were pooled in equimolar amounts and sequenced using 150-bp paired-end (PE) chemistry on an Illumina NovaSeq 6000 instrument. Following initial screening for library complexity, additional libraries were prepared using aliquots of the same DNA extract and then sequenced as described above.

#### Genomic data processing

Base calling was performed using Illumina *bcl2fastq2* conversion software. Only sequences with correct index combinations were retained. Fastq files were processed using *PALEOMIX v. 1.2.13.4*.^28^ Residual adapter sequences and low-quality reads (base quality Q<20) were removed using *AdapterRemoval v2.3.1*,^29^ retaining only reads ≥25 bp. Trimmed, filtered reads were aligned to NCBI build 38 of the human reference genome using the *Burrows-Wheeler Aligner*,^30^ as implemented by *BWA v. 0.7.10*, with the ‘backtrack’ algorithm and seeding disabled (*-l 1024*) to ensure higher sensitivity.^48^ The minimum base quality was set to 15, the maximum number of gap opens was set to 2, and *-n* was set to 0.01. Only mapped reads with Phred-scaled mapping quality (mapQ) scores ≥ 20 were kept. Base quality scores were rescaled with *MapDamage v. 2.0.9*^31^ to exclude likely-damaged bases. Mapped reads were filtered on a library-based level for PCR and optical duplicates using *PicardTools v. 2.21.3* (broadinstitute.github.io/picard). Read depth and coverage were determined using *BEDtools v. 2.29.2*^32^ as well as an in-house *python* script. Only mapped reads with Phred-scaled mapping and base quality scores ≥20 were imputed. Ancient genomes from previously published studies were processed in the same way.

#### Population genetic reference data

For the population genetic analyses, we used micro-array SNP genotype data from present-day populations of Iceland, England, Ireland, Wales, Scotland, Denmark, Sweden and Norway.^19^ To obtain diploid genotypes for the Well-man at the positions covered by the micro-array genotypes, we performed imputation as described below. For the sake of comparison with the Well-man, and to gain insight into the past population structure of Norway, we also examined the genomes of 23 previously published ancient individuals from central and northern Norway,^21^^,^^22^ selected on the basis of (1) being from the same time period as the Well-man, (2) having been excavated in central and north Norway with genomic analysis confirming this affinity, and (3) having an average nuclear genome sequencing depth of at least 0.2x. These genomes had a mean sequencing depth of 1.7x and all of them were imputed in the same way as the Well-man. We used these ancient individuals to determine whether the Well-man could be genetically distinguished from roughly contemporaneous ancient individuals from north and central Norway. Information about the ancient Norwegian genomes used in this analyses are presented in Table S5.

### Quantification and statistical analyses

#### Contamination estimates

Sample contamination was estimated using the mitochondrial genome, on the one hand, and X-chromosome heterozygosity, on the other. Each of the six libraries from the Well-man was tested individually for laboratory contamination using mapQ ≥ 30. Each library was tested with the contamMix contamination estimation method as described in.^49^ All libraries showed less than 4% contamination levels and were thus deemed appropriate for further analysis. The final BAM file generated from all the clean libraries was subjected to X-chromosome heterozygosity contamination analysis using a genotype-likelihood (option *-gl 1*) approach and two separate tests (‘Method 1’ and ‘Method 2’)^50^ as implemented in *ANGSD v.0.935*^33^ and the companion program *realSFS*. Here, we excluded sites with less than two reads and more than 10 reads, setting the region (option *-r*) to 5,000,000–154,900,000, ignoring transitions and masking regions of low genome accessibility as annotated by the 1000 Genomes Project (1KGP). The contamination test results are presented in Table S2.

#### Karyotype determination, uniparental markers, phenotypic appearance and inbreeding

Karyotype determination was performed as described in^13^ by calculating the ratio of reads aligning to the Y chromosome (*n*_*Y*_) as the fraction of total reads aligning to X and Y chromosome (*n*_*Y+X*_), denoted as *R*_*Y*_ = (*n*_*Y*_/*n*_*Y+X*_). Confidence intervals were calculated as *R*_*Y*_±1.96×*R*_*Y*_×(1-*R*_*Y*_)/*n*_*Y+X*_. We used *haploGrouper*^34^ with the International Society of Genetic Geneaology (ISOGG) 2019 Y-DNA Haplogroup Tree^27^ to assign Y-chromosomal haplogroup. The same approach was used to assign a mitochondrial haplogroup, based on the *PhyloTree* build 17 phylogeny of human mitochondrial DNA variation.^15^ To predict phenotypes related to skin, hair and eye colour, we used the algorithm at the HIrisPlex website (hirisplex.erasmusmc.nl), based on imputed genotypes at 41 informative sites specified there.^23^^,^^24^^,^^25^ The inbreeding coefficient for ancient individuals was estimated by identifying large chromosome fragments with an excess of homozygous imputed genotypes. Only 10,420,846 SNPs with MAF > 0.03 in the 1KGP data were used for this analysis. Considering each of these SNPs, ordered by chromosome and physical position, potential homozygous fragments were extended until one of two thresholds were violated: the number of heterozygous genotypes exceeded 60 or the proportion of heterozygous genotypes exceeded 1%. These parameter values were used to avoid overly conservative rejection of potential homozygous fragments due to genotype errors, which are more common in genotype data from ancient samples than present-day samples. When either threshold was violated or when reaching the end of a chromosome, the potential homozygous fragment was accepted only if it was longer than 1 cM and contained at least 1000 SNPs with homozygous genotypes. Finally, the inbreeding coefficient was estimated as the sum of accepted homozygous fragments identified on autosomal chromosomes as a proportion of the total length of the autosomal genome (3523.4 cM). The sex determination analysis results are presented in Table S2.

#### Imputation

Imputation was performed with *GLIMPSE,*^35^ using phased genotypes from the 1KGP as a reference panel (release 20201028_3202_phased within 1000G_2504_high_coverage). As *GLIMPSE* imputes only bi-allelic SNP loci, we first filtered and transformed the VCF files for the autosomal and X chromosomes, such that they contained only bi-allelic SNPs (*n* = 61,715,567). To make imputation more efficient and effective, we reduced the number of bi-allelic SNPs to 35,277,439, based on being also observed in at least one of three large whole-genome sequence data sets available at deCODE Genetics: 63,118 Icelanders, 25,215 individuals from Europe and the United States populations, and 150,119 individuals living in the British Isles.^51^ Genotype calling for the Well-man and other ancient individuals was performed using bcftools, following the *GLIMPSE* authors’ recommendations (odelaneau.github.io/GLIMPSE). We then used *GLIMPSE* to perform imputation and phasing for each chromosome in overlapping 2-Mbp sections.^35^ Results of the genomic imputation are presented in Table S6.

#### Population genetic analyses

All autosomal analyses of ancient individuals were based on imputed diploid genotypes. Principal components analysis (PCA) projection was performed using an in-house *python* script^19^ that applies the same approach as implemented in *lsqproject* from the software *smartpca*,^36^ based on eigenvector values derived from the application of smartpca to several different sets of genotyped individuals from present-day reference populations. This approach facilitated projection of individuals onto a previously calculated PCA and avoided the computationally intensive step of calculating new principal coordinates for each projection. The reference populations comprised (1) an extensive set of 9,052 individuals from 72 European populations, (2) a set of eight North Atlantic populations from Scandinavia, the British and Irish Isles, and Iceland,^19^ and (3) a set of 6,140 genotyped Norwegians with information about county of residence.^20^ We used the software *admixture*^52^ in supervised mode to estimate the Norse versus Gaelic (*K*=2) ancestry of the Well-man, based on training sets of 1,057 Norwegians and 1,117 Swedes for Norse, and 230 Irish and 99 Scots for Gaelic.^19^ Here the term “Norse” is a collective descriptor for people with most of their ancestry from Norway and Sweden, while the term “Gaelic” is a collective descriptor for people with most of their ancestry from Ireland and Scotland, both of which serve as proxies for people from those regions during the lifetime of the Well-man. The total number of present-day Norwegian genomes in our data set is 6,140 (Table S4). *f*_4_-statistics were calculated in the form YRI:Ancient;Danish:XXX, where Ancient represents one of the 22 whole genomes sequenced from ancient individuals. Results of the population genetic analyses are presented in Figures 2, 3, and 4A, and Table S4.

#### Pathogen screening

Data from the six libraries generated from tooth extracts were adapter-trimmed using *AdapterRemoval v. 2.3.1*,^29^ with overlapping read pairs collapsed if they overlapped by at least 11 bp. The trimmed reads were analysed with *MALT v. 0.4.1*^37^ using a custom database constructed using complete bacterial, viral and archaeal genomes as previously described,^53^ with the addition of a curated set of representative protist mitochondrial, kinetoplast and apicoplast genomes downloaded from Genbank (March 18, 2021). The *MALT* run was performed in *BlastN* mode with *SemiGlobal* alignment. The *--minPercentIdentity* parameter was set to 95, *--minSupport* was set to 10 and *--topPercent* to 1. Remaining parameters were set to their defaults. The *MALT* output rma6 files were subsequently screened for pathogens using the *MaltExtract* and *AMPS* tools integrated in the *HOPS* pipeline.^38^ A custom list of taxa (github.com/ashildv/custom_hops_pathogen_list_v4) was used. Additionally, the rma6 output files were visually inspected using *MEGAN6*.^39^ The negative results of the pathogen screening are presented in the Results text.

## Supplemental information

Document S1. Figures S1–S4 and Tables S1–S3 and S5–S7

Table S4. Euclidean distances from each ancient Norwegian genome to present-day Norwegian reference populations using the first ten principal components, related to Figure 4
