# Supplementary material for: Corroborating written history with ancient DNA: The case of the Well-man described in an Old Norse saga
Source: iScience. 2024 Oct 25;27(11):111076. doi: 10.1016/j.isci.2024.111076 (PMC11607536; doi:10.1016/j.isci.2024.111076)
Supplement: Document S1. Figures S1–S4 and Tables S1–S3 and S5–S7 [file mmc1.pdf]

iScience, Volume *27*

## Supplemental information

# Corroborating written history with ancient DNA: The case of the Well-man described in an Old Norse *saga*

**Martin R. Ellegaard, S. Sunna Ebenesersdóttir, Kristjan H.S. Moore, Anna Petersén, Åshild J. Vågene, Vanessa C. Bieker, Sean D. Denham, Gianpiero L. Cavalleri, Edmund Gilbert, Thomas Werge, Thomas F. Hansen, Ingrid Kockum, Lars Alfredsson, Tomas Olsson, Eivind Hovig, M. Thomas P. Gilbert, Kári Stefánsson, Hans K. Stenøien, Agnar Helgason, and Michael D. Martin**

**Supplementary Information**

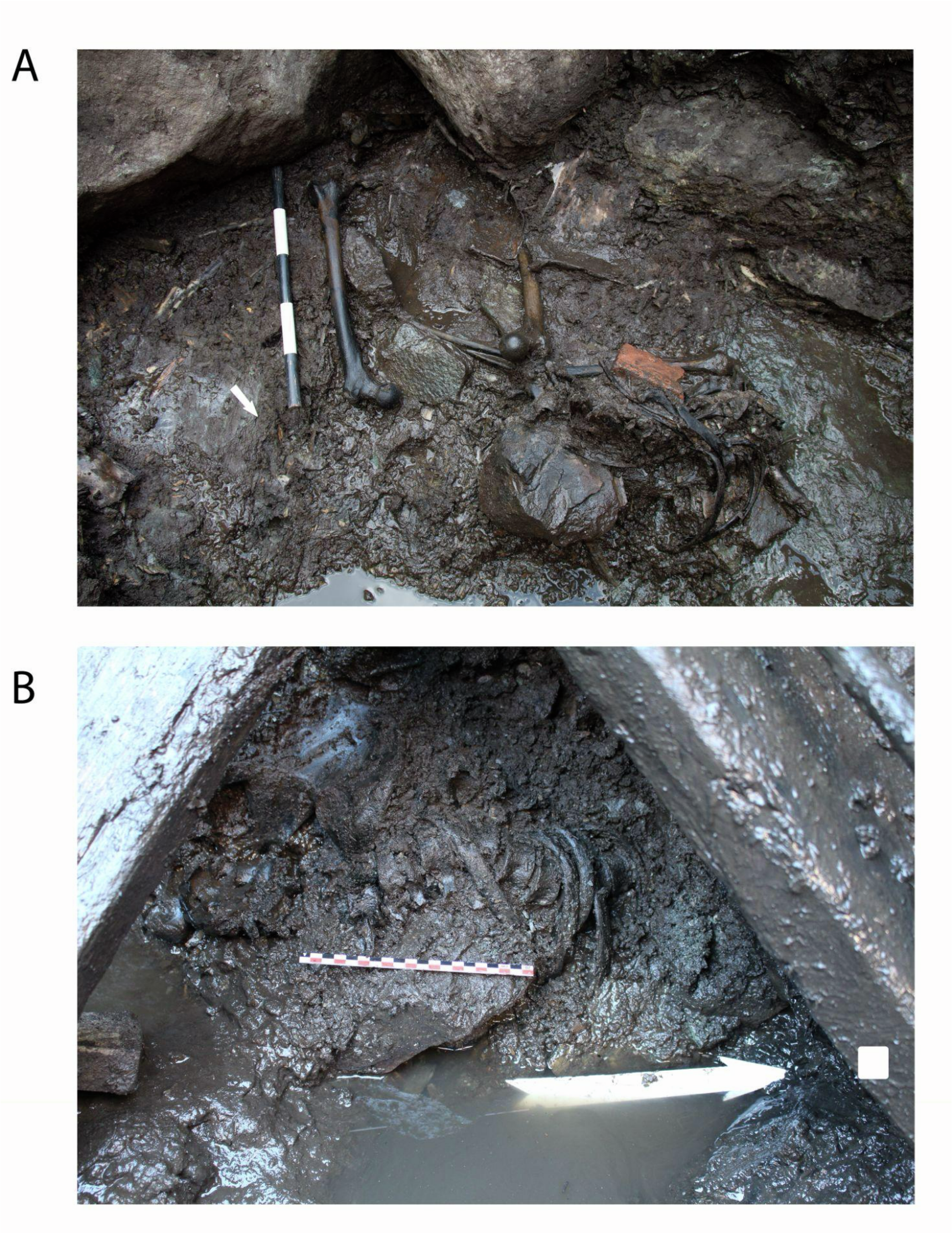


**Figure S1**. Photographs of the Well-man skeletal remains during excavation, Related to Figure 1. (A) Excavation in 2016. Image courtesy of NIKU. (B) Excavation in 2014. Image courtesy of NIKU.

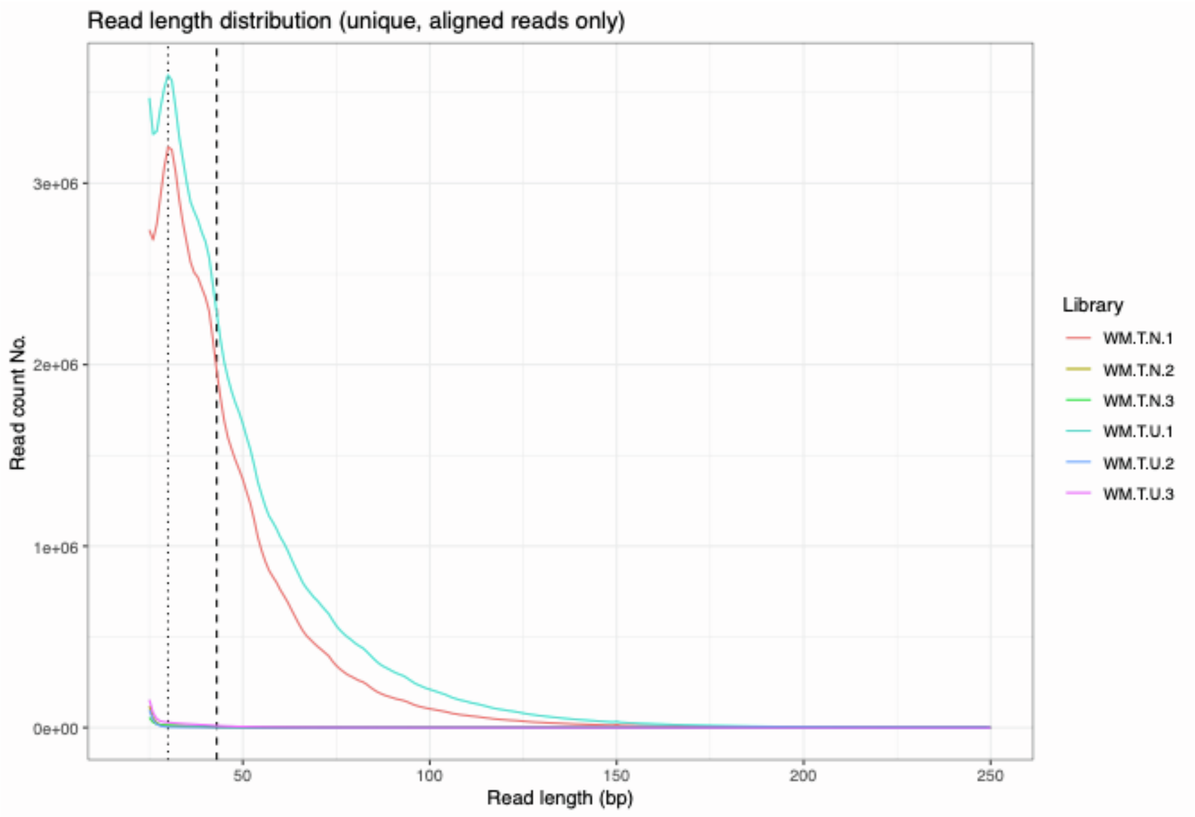


**Figure S2**. Distributions of read length for six libraries built on the Well-man DNA extracts, Related to Figure 1. The dotted line indicates the mode of all libraries. The dashed line indicates the mean of all libraries.

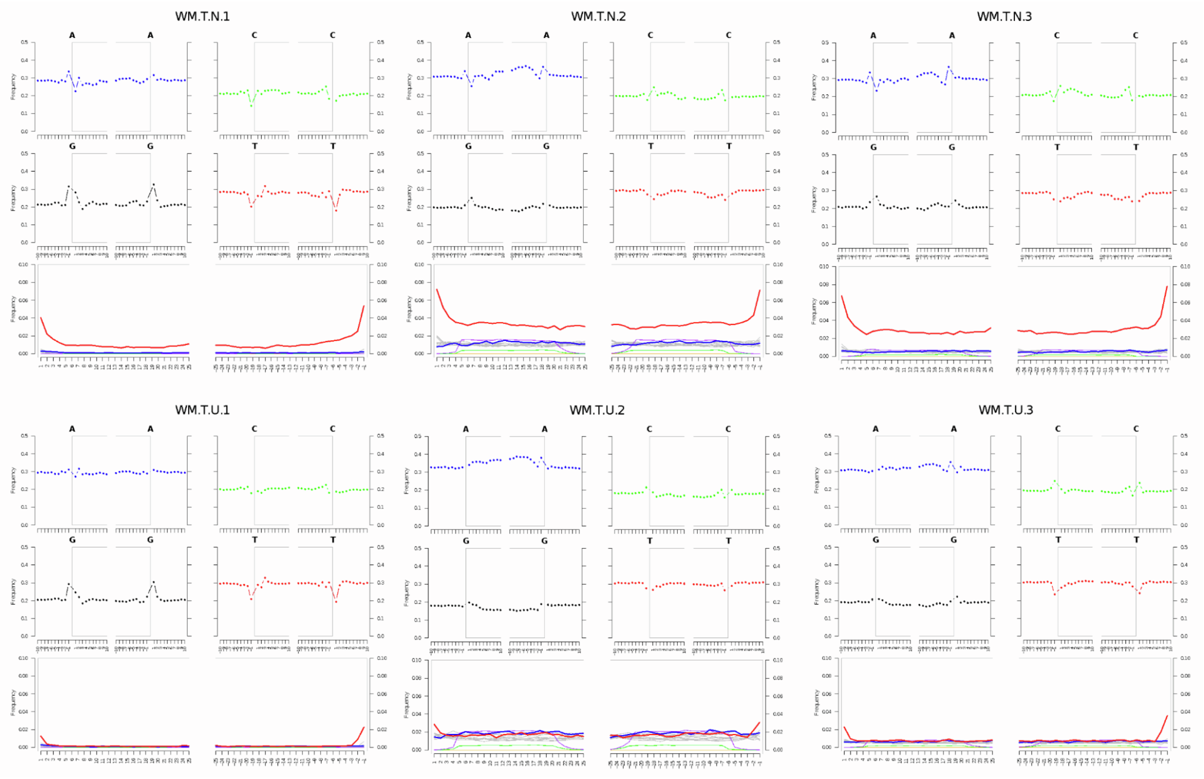


**Figure S3**. MapDamage results for the six Well-man libraries, Related to Figure 1. Top row: DNA extracts not USER-treated (samples WM.T.N.1, WM.T.N.2, and WM.T.N.3). Bottom row: USER-treated DNA extracts (samples WM.T.U.1, WM.T.U.2, and WM.T.U.3).

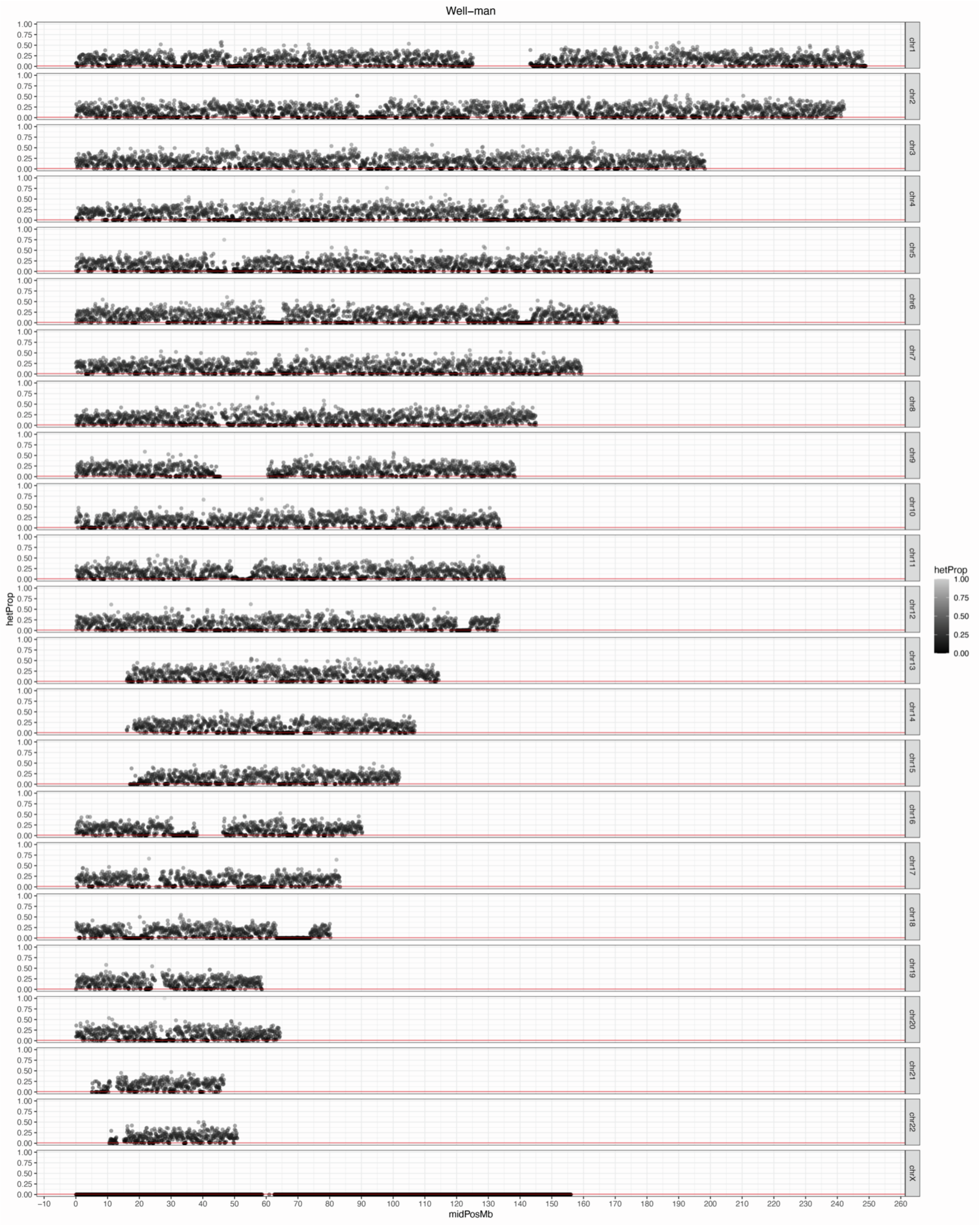


**Figure S4**. Chromosome-specific distributions of heterozygosity for the Well-man sample, Related to Figure 2.

**Table S1**. Data generation and mapping statistics, Related to Figure 1. Total read pairs: total number of 150-bp read pairs. Mapped reads: number of reads mapped to the human reference genome. Unique reads: number of reads uniquely mapped to the human reference genome. Mean read length: average fragment length of unique reads. Endog. content: proportion of uniquely mapped reads to total reads. Depth MT: final sequencing depth of mitochondrial genome after read filtering. Depth NR: final sequencing depth of autosomes. Y-chrom. hap.: Y-chromosome haplogroup. MT hap: mitochondrial genome haplogroup. Library NTC = library no-template control.

| Sample ID | Total read pairs | Mapped reads | Unique reads | Mean read length (bp) | Library clonality (%) | Endog. content (%) | Mean depth MT (x) | Mean depth AU (x) | 9 Y-chrom. hap. | 10 MT hap. |
|---|---|---|---|---|---|---|---|---|---|---|
| Well-man | 5,860,146,617 | 262,100,736 | 204,791,462 | 47.9 | 21.9 | 3.49 | 1278.7 | 3.18 | I1a1a2a1 | H2a2a1 |
| Extraction Blank | 2,064,161 | 428 | 100 | 109.8 | 76.6 | 0.02 | 0.0 | 0.00 | - | - |
| Library NTC | 11,066,006 | 6,235 | 3,076 | 74.8 | 50.7 | 0.06 | 0.0 | 0.00 | - | - |

**Table S2**. Estimates of contamination in each of the six Well-man libraries, Related to Figure 1. MT: mitochondrial genome.

| Library type | Test type | *ContamMix* | | | *ANGSD* | | | |
|---|---|---|---|---|---|---|---|---|
| | | Reads in MT | Better match | Contamination estimate (%) | Method1 | | Method2 | |
| WM.T.N.1 | MT | 26,856 | 867 | 3.2 | - | - | - | - |
| WM.T.N.2 | MT | 6,473 | 140 | 2.2 | - | - | - | - |
| WM.T.N.3 | MT | 4,292 | 87 | 2.0 | - | - | - | - |
| WM.T.U.1 | MT | 28,305 | 99 | 0.3 | - | - | - | - |
| WM.T.U.2 | MT | 4,268 | 7 | 0.2 | - | - | - | - |
| WM.T.U.3 | MT | 12,420 | 16 | 0.1 | - | - | - | - |
| all libraries | X-chrom | - | - | - | 0.00831% | *p*-val=0.6226 | 0.00663% | *p*-val=0.8815 |

**Table S3**. Well-man sex determination based on statistical analysis of reads mapping to the X and Y chromosomes, Related to Figure 1.

| Reads mapped to X | Reads mapped to Y | Total X+Y reads | Ry | SE | 95% CI | NGS sex (male / female) |
|---|---|---|---|---|---|---|
| 5,379,644 | 601,184 | 5,980,828 | 0.100519 | 0.000123 | 0.000241 | male |

**Table S5**. Provenance and information on sex, contamination, and haplogroup for 24 ancient Norwegian specimens analysed in this study, Related to Figure 4.

| Sample ID | Burial region | Lat | Lon | Age | Sample type | Depth (X) | Sex | Contam. mtDNA (%) | Y hap. | mtDNA hap. | Publication |
|---|---|---|---|---|---|---|---|---|---|---|---|
| SK312 | Trondheim | 63.4 | 10.4 | 1100-1175 CE | Tooth | 0.46 | F | 1.45 | N/A | U5a2d1 | Gopalakrishnan et al. (2022) |
| SK340 | Trondheim | 63.4 | 10.4 | 1100-1175 CE | Tooth | 1.09 | F | 1.35 | N/A | T2e1 | Gopalakrishnan et al. (2022) |
| SK356 | Trondheim | 63.4 | 10.4 | 1175-1275 CE | Tooth | 7.15 | F | 0 | N/A | H10a1 | Gopalakrishnan et al. (2022) |
| VK387 | Oppland | 60.31 | 10.43 | 900-1100 CE | Petrous | 1.20 | F | 1.56 | N/A | U5a1a1 | Margaryan et al. 2020 |
| VK388 | Nordland | 68.34 | 15.66 | 700-1500 CE | Tooth | 1.01 | M | 0.2 | I1 | J1c5 | Margaryan et al. 2020 |
| VK389 | Telemark | 59.22 | 9.61 | 900-1000 CE | Petrous | 1.29 | M | 0.82 | R1b1a1b1a1a1c2b2a1b1a4b | T2b | Margaryan et al. 2020 |
| VK392 | Telemark | 59.58 | 7.92 | 700-1000 CE | Tooth | 1.14 | F | 1.92 | N/A | H1g1 | Margaryan et al. 2020 |
| VK393 | Hedmark | 60.84 | 11 | 900-1000 CE | Tooth | 1.25 | F | 1.04 | N/A | U8a1a | Margaryan et al. 2020 |
| VK394 | Hedmark | | 11.34 | 900-1000 CE | Petrous | 0.95 | M | 0.61 | R1a1a1b1a3 | H13a1a1a | Margaryan et al. 2020 |
| VK414 | Oppland | 61.88 | 8.4 | 900-1100 CE | Petrous | 1.32 | M | 1.35 | R1a1a1b1a3a2a | H6a1a | Margaryan et al. 2020 |
| VK415 | Oppland | 60.29 | 10.63 | 700-1100 CE | Petrous | 1.31 | F | 0.18 | N/A | H6a1a | Margaryan et al. 2020 |
| VK417 | Oppland | 60.36 | 10.57 | 600-1100 CE | Petrous | 1.25 | F | 0.55 | N/A | H7b | Margaryan et al. 2020 |
| VK419 | Nordland | 67.29 | 14.42 | 600-1000 CE | Petrous | 1.25 | M | 1.82 | N1a1a1a1a1a | U5b1b1g1 | Margaryan et al. 2020 |
| VK420 | Hedmark | 60.8 | 11.1 | 700-1100 | Petrous | 1.52 | M | 0.62 | I1 | I4a | Margaryan et al. 2020 |
| VK514 | Nordland | 67.91 | 15.13 | 600-1000 CE | Tooth | 1.11 | M | 1.3 | R1a1a1b1a3a1 | K2b1a1 | Margaryan et al. 2020 |
| VK516 | Sor-Trondelag | 63.7 | 9.75 | 900-1000 CE | Tooth | 1.44 | M | 0.87 | R1a1a1b1a3a2 | H6a1a | Margaryan et al. 2020 |
| VK519 | Nordland | 67.93 | 14.96 | 600-1000 CE | Tooth | 0.24 | M | 0.3 | I1 | HV0a1 | Margaryan et al. 2020 |
| VK520 | Troms | 69.65 | 18.13 | 900-1000 CE | Tooth | 1.84 | F | 0.67 | N/A | U5b2a1a2 | Margaryan et al. 2020 |
| VK523 | Sor-Trondelag | 63.83 | 9.7 | 500-1100 CE | Petrous | 1.15 | F | 0.86 | N/A | I1a1 | Margaryan et al. 2020 |

| | | | | | | | | | | | |
|---|---|---|---|---|---|---|---|---|---|---|---|
| VK524 | Nordland | 66.26 | 12.73 | 900-1000 CE | Tooth | 1.49 | M | 0.45 | I1a2a1a1d1a | HV0a1 | Margaryan et al. 2020 |
| VK529 | Nordland | 67.64 | 14.79 | 700-900 CE | Tooth | 2.02 | M | 1.11 | I1a1b1a4a2 | H7 | Margaryan et al. 2020 |
| VK547 | Nordland | 68.55 | 16.4 | 800-1100 CE | Tooth | 5.39 | M | 0.74 | I1a1b1a4a2 | V | Margaryan et al. 2020 |
| VK548 | Nord-Trondelag | 63.44 | 11.22 | 900-1000 CE | Tooth | 3.80 | F | 0.47 | N/A | A12a | Margaryan et al. 2020 |
| Well-man | Trondheim | 63.25 | 10.21 | 1154-1285 CE | Tooth | 3.40 | M | 1.34 | I1a1a2a1 | H2a2a1 | This study |

**Table S6**. Heterozygosity proportions and related statistics in the autosomes of ancient Norwegian individuals, Related to Figure 4. Hom GT cnt: number of homozygous genotypes. Het GT cnt: number of homozygous genotypes. Miss GT cnt: number of missing genotypes. Het prop: proportion of genotypes in heterozygous state. Frag cnt: number of autosomal chromosome regions in contiguous homozygous state. Frag len pos sum: total length of autosomal chromosome regions in contiguous homozygous state (bp). Frag len cM sum: total length of autosomal chromosome regions in contiguous homozygous state (cM). Hom IBD prop: inbreeding coefficient (proportion of autosomal genome identical by descent).

| Sample | Hom GT cnt | Het GT cnt | Missing GT cnt | Het prop | Frag cnt | Frag len pos sum | Frag len cM sum | Hom IBD prop |
|---|---|---|---|---|---|---|---|---|
| SK312 | 10,438,873 | 2,034,685 | 0 | 0.1631200 | 1 | 1,812,200 | 1.371 | 0.000389 |
| SK340 | 10,410,684 | 2,062,874 | 0 | 0.1653800 | 2 | 4,043,691 | 6.413 | 0.00182 |
| VK519 | 10,406,730 | 2,066,828 | 0 | 0.1656970 | 2 | 4,074,100 | 3.232 | 0.000917 |
| VK420 | 10,444,151 | 2,029,407 | 0 | 0.1626970 | 3 | 4,316,013 | 5.894 | 0.001673 |
| VK415 | 10,424,151 | 2,049,407 | 0 | 0.1643000 | 3 | 5,802,075 | 7.602 | 0.002157 |
| VK419 | 10,428,162 | 2,045,396 | 0 | 0.1639790 | 3 | 6,074,603 | 6.27 | 0.001779 |
| VK524 | 10,420,793 | 2,052,765 | 0 | 0.1645690 | 5 | 7,826,070 | 7.252 | 0.002058 |
| VK514 | 10,430,987 | 2,042,571 | 0 | 0.1637520 | 3 | 8,211,893 | 5.652 | 0.001604 |
| SK356 | 10,432,951 | 2,040,607 | 0 | 0.1635950 | 2 | 8,256,441 | 2.627 | 0.000746 |
| VK388 | 10,428,130 | 2,045,428 | 0 | 0.1639810 | 5 | 9,048,710 | 8.437 | 0.002395 |
| VK394 | 10,420,098 | 2,053,460 | 0 | 0.1646250 | 3 | 9,371,627 | 5.19 | 0.001473 |
| VK547 | 10,406,135 | 2,067,423 | 0 | 0.1657440 | 4 | 9,780,076 | 13.357 | 0.003791 |
| VK548 | 10,429,889 | 2,043,669 | 0 | 0.1638400 | 6 | 11,722,002 | 13.698 | 0.003888 |
| VK387 | 10,412,174 | 2,061,384 | 0 | 0.1652600 | 6 | 11,970,757 | 13.379 | 0.003797 |
| VK414 | 10,423,959 | 2,049,599 | 0 | 0.1643160 | 7 | 13,523,934 | 18.027 | 0.005116 |
| VK520 | 10,425,717 | 2,047,841 | 0 | 0.1641750 | 5 | 14,002,723 | 13.101 | 0.003718 |
| VK389 | 10,408,052 | 2,065,506 | 0 | 0.1655910 | 6 | 17,949,501 | 9.986 | 0.002834 |
| VK516 | 10,445,104 | 2,028,454 | 0 | 0.1626200 | 9 | 18,230,084 | 27.7 | 0.007862 |
| VK529 | 10,422,114 | 2,051,444 | 0 | 0.1644630 | 6 | 18,639,719 | 9.394 | 0.002666 |
| VK393 | 10,428,235 | 2,045,323 | 0 | 0.1639730 | 9 | 19,103,449 | 22.262 | 0.006318 |
| Well-man | 10,420,846 | 2,052,712 | 0 | 0.1645650 | 10 | 29,563,116 | 40.223 | 0.011416 |
| VK392 | 10,449,351 | 2,024,207 | 0 | 0.1622800 | 10 | 31,431,160 | 29.791 | 0.008455 |
| VK417 | 10,456,171 | 2,017,387 | 0 | 0.1617330 | 15 | 57,554,666 | 70.963 | 0.02014 |
| VK523 | 10,483,493 | 1,990,065 | 0 | 0.1595430 | 18 | 61,872,880 | 68.996 | 0.019582 |

**Table S7**. HIrisPlex pigmentation phenotype allele dosages for the Well-man, Related to Figure 4.

| Variant | Allele dosage in Well-man | | Variant | Allele dosage in Well-man |
|---|---|---|---|---|
| rs312262906_A | 0 | | Full_AUC_BlueEye | 0.93897364 |
| rs11547464_A | 0 | | Full_AUC_IntermediateEye | 0.73608861 |
| rs885479_T | 0 | | Full_AUC_BrownEye | 0.94607779 |
| rs1805008_T | 1 | | Numb_missingSNPs_Eye | 0 |
| rs1805005_T | 0 | | AUC_Loss_BlueEye | 0 |
| rs1805006_A | 0 | | AUC_Loss_IntermediateEye | 0 |
| rs1805007_T | 0 | | AUC_Loss_BrownEye | 0 |
| rs1805009_C | 0 | | PBlondHair | 0.38480042 |
| rs201326893_A | NA | | PBrownHair | 0.41050149 |
| rs2228479_A | 0 | | PRedHair | 0.16092068 |
| rs1110400_C | 0 | | PBlackHair | 0.04377742 |
| rs28777_C | 0 | | Full_AUC_BlondHair | 0.81328159 |
| rs16891982_C | 0 | | Full_AUC_BrownHair | 0.74106545 |
| rs12821256_G | 0 | | Full_AUC_RedHair | 0.92893302 |
| rs4959270_A | 1 | | Full_AUC_BlackHair | 0.85924813 |
| rs12203592_T | 1 | | Numb_missingSNPs_Hair | 1 |
| rs1042602_T | 0 | | Name_missingSNPs_Hair | rs201326893_A |
| rs1800407_A | 0 | | AUC_Loss_BlondHair | 0.00140796 |
| rs2402130_G | 0 | | AUC_Loss_BrownHair | 0.00042713 |
| rs12913832_T | 0 | | AUC_Loss_RedHair | 0.00332208 |
| rs2378249_C | 1 | | AUC_Loss_BlackHair | 3.63E-05 |
| rs12896399_T | 1 | | PLightHair | 8.69E-01 |
| rs1393350_T | 0 | | PDarkHair | 0.13086645 |
| rs683_G | 1 | | Full_AUC_HairShade | 0.90544361 |
| rs3114908_T | 0 | | Numb_missingSNPs_HairShade | 0 |
| rs1800414_C | 0 | | AUC_Loss_HairShade | 0 |
| rs10756819_G | 2 | | PVeryPaleSkin | 9.19E-08 |
| rs2238289_C | 0 | | PPaleSkin | 1.62E-09 |
| rs17128291_C | 1 | | PIntermediateSkin | 1.00E+00 |
| rs6497292_C | 0 | | PDarkSkin | 1.49E-19 |
| rs1129038_G | 0 | | PDarktoBlackSkin | 7.62E-13 |
| rs1667394_C | 1 | | Full_AUC_Very PaleSkin | 8.30E-01 |
| rs1126809_A | 0 | | Full_AUC_PaleSkin | 0.76279635 |
| rs1470608_A | 0 | | Full_AUC_IntermediateSkin | 0.78330427 |
| rs1426654_G | 1 | | Full_AUC_DarkSkin | 0.9806167 |
| rs6119471_C | 2 | | Full_AUC_DarktoBlackSkin | 0.99347337 |
| rs1545397_T | 0 | | Numb_missingSNPs_Skin | 0 |
| rs6059655_T | 1 | | AUC_Loss_VeryPaleSkin | 0 |
| rs12441727_A | 0 | | AUC_Loss_PaleSkin | 0 |
| rs3212355_A | 0 | | AUC_Loss_IntermediateSkin | 0 |
| rs8051733_C | 1 | | AUC_Loss_DarkSkin | 0 |
| PBlueEye | 0.92584671 | | AUC_Loss_DarktoBlackSkin | 0 |
| PIntermediateEye | 0.05707147 | | | |
| PBrownEye | 0.01708183 | | | |
